# Neutralization of excessive CCL28 improves wound healing in diabetic mice

**DOI:** 10.3389/fphar.2023.1087924

**Published:** 2023-01-13

**Authors:** Zhenlong Chen, Jacob M. Haus, Luisa A. DiPietro, Timothy J. Koh, Richard D. Minshall

**Affiliations:** ^1^ Department of Anesthesiology, College of Medicine, University of Illinois at Chicago, Chicago, IL, United States; ^2^ School of Kinesiology, University of Michigan, Ann Arbor, MI, United States; ^3^ Center for Wound Healing and Tissue Regeneration, College of Dentistry, University of Illinois at Chicago, Chicago, IL, United States; ^4^ Department of Periodontics, College of Dentistry, University of Illinois at Chicago, Chicago, IL, United States; ^5^ Department of Kinesiology and Nutrition, College of Applied Health Sciences, University of Illinois at Chicago, Chicago, IL, United States; ^6^ Department of Pharmacology and Regenerative Medicine, College of Medicine, University of Illinois at Chicago, Chicago, IL, United States

**Keywords:** CCL28, CCR10, diabetic foot ulcers, eNOS, angiogenesis

## Abstract

**Introduction:** Chronic, non-healing skin wounds such as diabetic foot ulcers (DFUs) are common in patients with type 2 diabetes mellitus (T2DM) and often result in limb amputation and even death. However, mechanisms by which T2DM and inflammation negatively impact skin wound healing remains poorly understood. Here we investigate a mechanism by which an excessive level of chemokine CCL28, through its receptor CCR10, impairs wound healing in patients and mice with T2DM.

**Methods & Results:** Firstly, a higher level of CCL28 was observed in skin and plasma in both patients with T2DM, and in obesity-induced type 2 diabetic *db/db* mice. Compared with *WT* mice, adipose tissue from *db/db* mice released 50% more CCL28, as well as 2- to 3-fold more IL-1β, IL-6, and TNF-α, and less VEGF, as determined by ELISA measurements. Secondly, overexpression of CCL28 with adenovirus (Adv-CCL28) caused elevation of proinflammatory cytokines as well as CCR10 expression and also reduced eNOS expression in the dorsal skin of WT mice as compared with control Adv. Thirdly, topical application of neutralizing anti-CCL28 Ab dose-dependently accelerated wound closure and eNOS expression, and decreased IL-6 level, with an optimal dose of 1 μg/wound. In addition, mRNA levels of eNOS and anti-inflammatory cytokine IL-4 were increased as shown by real-time RT-PCR. The interaction between eNOS and CCR10 was significantly reduced in diabetic mouse wounds following application of the optimal dose of anti-CCL28 Ab, and eNOS expression increased. Finally, enhanced VEGF production and increased subdermal vessel density as indicated by CD31 immunostaining were also observed with anti-CCL28 Ab.

**Discussion:** Taken together, topical application of neutralizing anti-CCL28 Ab improved dorsal skin wound healing by reducing CCR10 activation and inflammation in part by preventing eNOS downregulation, increasing VEGF production, and restoring angiogenesis. These results indicate anti-CCL28 Ab has significant potential as a therapeutic strategy for treatment of chronic non-healing diabetic skin wounds such as DFUs.

## Introduction

Type 2 diabetes mellitus (T2DM) is now the most common metabolic disorder and third leading cause of death in the US (Stokes and Preston, 2017). Cardiovascular complications of T2DM greatly reduce the quality of life. In 2015, 415 million people globally were diagnosed with T2DM, and this number is projected to rise to an alarming 642 million people by 2040 (International Diabetes Federation, 2015). Diabetic patients have a 25% lifetime risk of developing diabetic foot ulcers (DFUs) which precede amputation in up to 90% of cases (Ahmad et al., 2014; Hoffstad et al., 2015). Limb amputation significantly decreases the quality of life and contributes to the high morbidity, mortality (Schmidt et al., 2022), and healthcare costs associated with T2DM (Sinha et al., 2011). Five-year mortality rates after new-onset diabetic ulceration are between 43% and 74% for patients with lower-extremity amputation (Robbins et al., 2008).

Persistent hyperglycemia in diabetics leads to endothelial cell (EC) dysfunction which results in reduced pro-angiogenic signaling and NO production (Förstermann and Münze, 2006; Catrina and Zheng, 2016) in both macro- and microvascular beds in animals and human subjects (Candido et al., 2009; Ceriello, 2010). Reduced expression of endothelial nitric oxide synthase (eNOS) was identified as a major contributing factor of endothelial dysfunction and impaired blood flow to the extremities (Veves et al., 1998). We also observed less eNOS expression in subdermal biopsies from patients with T2DM (Mahmoud et al., 2016; Chen et al., 2018) and recently reported that chemokine receptor CCR10 activation by ligand CCL28 leads to a decrease in eNOS expression and NO production (Chen et al., 2020) resulting in defective angiogenesis in diabetic mice (Chen et al., 2022). Furthermore, topical application of myristoylated CCR10 binding domain 7 amino acid (Myr-CBD7) peptide prevented CCR10-eNOS interaction and subsequent eNOS downregulation, enhanced eNOS/NO levels, and improved wound healing in obesity-induced diabetic mice (Chen et al., 2022).

Chemokine CCL28, also known as Mucosae-associated Epithelial Chemokine (MEC), is constitutively produced in mucosal tissues including the digestive system, respiratory tract, and female reproductive system (Mohan et al., 2017). CCL28 was discovered (Wang et al., 2000) as a ligand for chemokine receptor CCR10 (Marchese et al., 1994). Both human and mouse CCL28 induce calcium mobilization in CCR10-expressing BAF/3 cells (Wang et al., 2000). CCL28 was also reported to induce the migration of IgA Ab-secreting cells (ASCs) *via* CCR10 and exhibit potent antimicrobial activity in the colon, whereas altered microbiota was observed in the colon in CCL28-deficient mice (Matsuo et al., 2018). CCL28 was also shown to be notably involved in the immune response to mucosal pathogens by regulating neutrophil recruitment and activation, and CCL28 knockout mice were highly susceptible to *Salmonella* infection in the gut (Burkhardt et al., 2019). The level of CCL28 in epithelial cells is modulated by pro-inflammatory cytokines and bacterial products indicating that it may play a role in attracting CCR10 + cells to the site of colonic inflammation (Hieshima et al., 2003; Ogawa et al., 2004). A previous study demonstrated that elevation of CCL28 facilitated homing of Tregs that foster ovarian tumor angiogenesis (Facciabene et al., 2011). Similar to these findings, we previously reported that overexpression of CCL28/CCR10 in synovial tissue was associated with upregulation of angiogenesis in patients with rheumatoid arthritis (Chen et al., 2015).

Previously, we observed an increase in the level of CCL28 and activation of its receptor CCR10 in diabetic skin resulting in internalization of the receptor and associated eNOS leading to eNOS degradation and delayed wound healing. Importantly, we observed an increase in eNOS/NO, reduction in inflammatory cytokine levels, and improvement in angiogenesis upon disruption of CCR10-eNOS interaction with a cell permeable 7 aa peptide derived from eNOS (Chen et al., 2022). As CCL28 at the higher doses observed in T2DM may induce signaling in non-endothelial cells in the wound environment *via* CCR10 as well as CCR3 receptors, we sought to assess the effect of CCL28 overexpression in WT mice as well as CCL28 neutralization in diabetic skin wounds with a specific antibody (Ab). Treatment with the optimum dose of 1 μg/ml anti-CCL28 Ab reduced proinflammatory cytokines, enhanced eNOS/NO levels, and stimulated angiogenesis, thus improving wound healing. On the contrary, overexpression of CCL28 in WT mice by injecting CCL28 adenovirus into skin wounds reduced NO production, increased cytokine levels, and attenuated wound healing. Taken together, these data suggest anti-CCL28 Ab may be a novel therapeutic strategy for treatment of DFUs.

## Methods

### Human subjects

Full experimental procedures have been presented previously (Mahmoud et al., 2016; Mey et al., 2018; Chen et al., 2022). Briefly, two groups of participants were recruited from the Chicago, IL, metropolitan area, a type 2 diabetes mellitus (T2DM) group (*n* = 8) and a lean healthy control (LHC) group (*n* = 10). Following an approximate 12 h overnight fast, blood samples were acquired *via* antecubital vein and subdermal needle biopsies of the *vastus lateralis* were obtained under local anesthesia (Lidocaine HCl 2%) using a 5 mm Bergstrom cannula with suction. All studies were approved by the Institutional Review Board of the University of Illinois at Chicago and performed in accordance with the Declaration of Helsinki. Written informed consent was obtained from all research participants during the initial screening visit.

### Mouse wound healing models

All animal procedures were performed in accordance with the Guide for the Care and Use of Laboratory Animals (National Institutes of Health) and as approved by the Institutional Animal Care and Use Committee. *WT* (C57BL/6J), *Lepr* ^*db+/db+*^(C57BLKS/J leptin receptor mutant strain, or *db/db*) exhibit an obesity-induced type 2 diabetes phenotype with total body weight of over 40 g, insulin resistance, and blood sugar level over 500 mg/dL by 9 weeks of age, which are nearly double that of *WT* littermate controls; Chen et al., 2022), and *eNOS* ^*−/−*^ mice were purchased from Jackson Laboratory (Bar Harbor, ME). Four 5 mm full thickness excisional wounds were made on the dorsal skin of mice (male, 9 weeks old) using a standard skin biopsy punch (Acuderm Inc. Fort Lauderdale, FL) under ketamine (100 mg/kg) and xylazine (5 mg/kg) anesthesia. 30 μl of IgG or neutralizing anti-CCL28 antibody (Ab) at indicated concentrations were applied topically to wounds in F-127 Pluronic gel (Sigma, St. Louis, MO; 25% gel in saline) immediately after wounding (Mirza et al., 2009). Wound size was determined as previously described (Zhao et al., 2016; Chen et al., 2020) and wound tissues were collected at indicated times for biochemical or histological analysis.

### Reagents

F-127 Pluronic gel, DTT, BSA, and RIPA buffer were from Sigma (St. Louis, MO). n-Octyl-β-d-glucopyranoside (ODG) was from RPI Corp (Mt Prospect, IL). Mouse neutralizing anti-CCL28 antibody and mouse ELISA kits were purchased from R&D Systems (Minneapolis, MN). Mouse anti-CCR10 and mouse anti-eNOS antibodies were from Santa Cruz Biotechnology (Dallas, TX). 4′,6-Diamidino-2-phenylindole (DAPI), fluorescently labeled secondary antibodies, Trizol and SYBR Green PCR mix were from ThermoFisher Scientific (Waltham, MA). Mouse anti-eNOS and mouse anti-actin antibodies were from BD Biosciences (San Diego, CA). Rabbit monoclonal anti-CD31 and Griess Reagent kit were from Cell Signaling Technology (Danvers, MA). Nitrocellulose membrane was from Bio-Rad Laboratories (Hercules, CA). SuperSignal West Femto Kit and Restore Western Stripping buffer were from Pierce (Rockford, IL). Skin punch biopsy tool was from Acuderm Inc. (Fort Lauderdale, FL). Cell strainer was from VWR Scientific (Franklin, MA). Control and CCL28 adenoviral expression vectors (Adv-Ctl, Adv-CCL28) were generated by Dr. Jody L. Martin (UIC Vector Core and Department of Pharmacology, University of California, Davis).

### Overexpression of adenovirus CCL28 (Adv-CCL28) in dorsal skin of WT mice

After shaving WT C57BL/6 mice (9 weeks old), 100 µl Adv-CCL28 or Adv-Ctl (1 × 10^9^ particles/ml) was subcutaneously injected into 4 labeled areas of dorsal skin per mouse. Five mm wounds were produced at each labeled area 21 days after Adv injection. Wound sizes were measured daily as described previously (Zhao et al., 2016; Chen et al., 2020), and then wounds were collected on day 7 and subsequently prepared for further analysis.

### ELISA

Human plasma, mouse skin wounds, or blood serum were collected, lysed, and prepared for ELISA measurements of mouse CCL28, IL-6, TNF-α, IL-1β and VEGF according to manufacturer’s instructions. For subcutaneous adipose tissue, the tissue was collected and minced. EBM-2 (without FBS) was added at a ratio of 3:1 (v/w) and the tissue was incubated for 24 h at 37°C. The conditioned medium was collected and filtered through a 70 μm cell strainer for ELISA measurements.

### Real-time RT-PCR

Total cellular RNA from mouse skin wounds and human tissue (collection of LHC and T2DM patient samples are described in Methods 2.1) was extracted using TRIzol. mRNA level used to assess relative gene expression was examined by real-time RT-PCR, employing a SYBR Green PCR mix as described previously (Chen et al., 2022). All primers were purchased from Integrated DNA Technologies IDT (Coralville, IA). Relative gene expression was determined by the ΔΔ*C*_*t*_ method based on GAPDH levels as described previously (Chen et al., 2013).

### Western blotting and co-immunoprecipitation (co-IP)

Dorsal skin or wounds were collected at indicated times and lysed in RIPA buffer for Western blotting. For Co-IP experiments, samples were lysed in 2% ODG (Tris buffer) as described previously (Chen et al., 2012; Chen et al., 2018).

### Hematoxylin and eosin staining and immunohistochemistry of skin wounds

For H&E staining, slides with 5 μm tissue sections were baked at 60°C for 30 min and stained with an Autostainer XL (Leica Microsystems, Wetzlar, Germany) using an established protocol (Chen et al., 2020; Chen et al., 2022). Immunohistochemistry of CD31 was performed in skin wound sections of *db/db* mice (6 mice per group) as described previously (Chen et al., 2022). Briefly, for wound tissue analysis of CD31 immunostaining, formalin-fixed paraffin-embedded sections were probed with a 1:50 dilution of anti-CD31 Ab overnight at 4°C, and then 1 h at room temperature with 1:500 dilution of a secondary antibody conjugated with Alexa-555. Images were obtained with a Zeiss LSM 880 confocal microscope. Vascular density, determined from 6 high-power fields per section and represented as a percentage of vascular tissue area occupied by CD31 relative to the total area of the field of view, was analyzed using Zeiss Zen and ImageJ software as described (Chen et al., 2022).

### Statistical analysis

All data were analyzed using the unpaired Student’s t-test or one-way ANOVA, followed by *post hoc* Tukey test. Unpaired Student’s t-test was used to compare two groups. If more than two groups were analyzed, one-way ANOVA followed by Tukey test was used. All data are expressed as mean ± SEM. Statistical significance was considered at *p* < .05.

## Results

### Elevated CCL28 in plasma and tissues in patients with type 2 diabetes mellitus (T2DM) and in obesity-induced diabetic *db/db* mice

**FIGURE 1:**
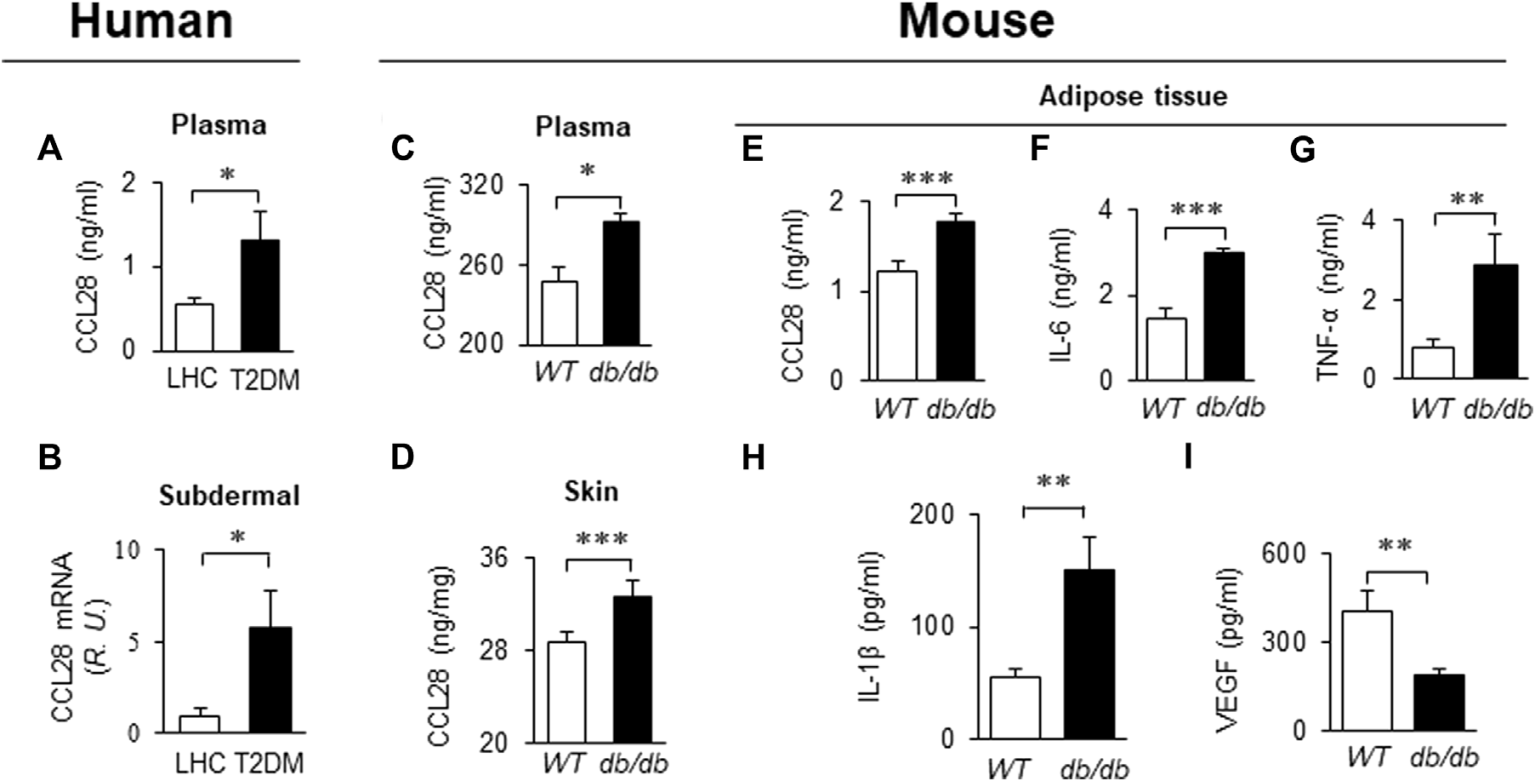
Elevated levels of CCL28 and proinflammatory cytokines in plasma and punch biopsies from human subjects and diabetic *db/db* mice. **(A)** CCL28 levels in plasma **(A)** and subdermal biopsies **(B)** obtained from lean healthy control (LHC) donors and patients with type 2 diabetes mellitus (T2DM). **(B)** Quantitative real-time RT-PCR revealed upregulated mRNA level of CCL28 in patients with T2DM compared to LHC donors. Values are mean ± SEM, n = 8–10 (*, *p* < .05). **(C)** Elevated level of CCL28 in the plasma as determined by ELISA, and **(D)** increased CCL28 mRNA in dorsal skin by real-time RT-PCR in *db/db* mice, as compared to WT mice. Values are mean ± SEM, n = 3–6 (**, *p* < .01, ***, *p* < .001). Elevated levels of CCL28 **(E)**, IL-6 **(F)**, TNF-α **(G)** and IL-1β **(H)**, and reduced VEGF level **(I)** by ELISA of culture medium from subcutaneous *db/db* adipose tissue as compared to WT mouse adipose. Values are mean ± SEM, n = 8 (**, *p* < .01, ***, *p* < .001).

Recently we reported that levels of CCL28 and CCR10 were significantly higher than CCL27 and CCR3 in the dorsal skin of diabetic mice. Thus, the CCL28/CCR10 chemokine signaling axis was chosen for further study (Chen et al., 2022). We observed an elevated level of CCL28 in plasma (Figure 1A) and subdermal biopsies *from the vastus lateralis* (Figure 1B) in patients with T2DM, compared to lean healthy control (LHC) donors. Similar to human samples, the level of CCL28 was significantly higher in plasma (Figure 1C) and dorsal skin (Figure 1D) in obese and diabetic *db/db* mice, as compared to wild type (*WT*) C57BL/6 mice. Previously, we reported a reduction in eNOS expression (Mahmoud et al., 2016; Chen et al., 2018) and excessive levels of CCL28/CCR10 in both patients with T2DM and diabetic mice (Chen et al., 2022). We next sought to establish a correlation between plasma and adipose tissue CCL28 levels in T2DM, as compared to *WT* mice. We observed an elevated level of CCL28 (by 50%; Figure 1E), IL-6 (one-fold; Figure 1F), TNF-α (2.5-fold; Figure 1G), and IL-6 (1.7-fold; Figure 1H), while VEGF level was reduced by about 50% (Figure 1I), as determined by ELISA of conditioned medium from *db/db* subcutaneous adipose tissue.

### Overexpression of CCL28 in dorsal skin of *WT* mice augmented levels of proinflammatory cytokines and reduced eNOS expression

**FIGURE 2:**
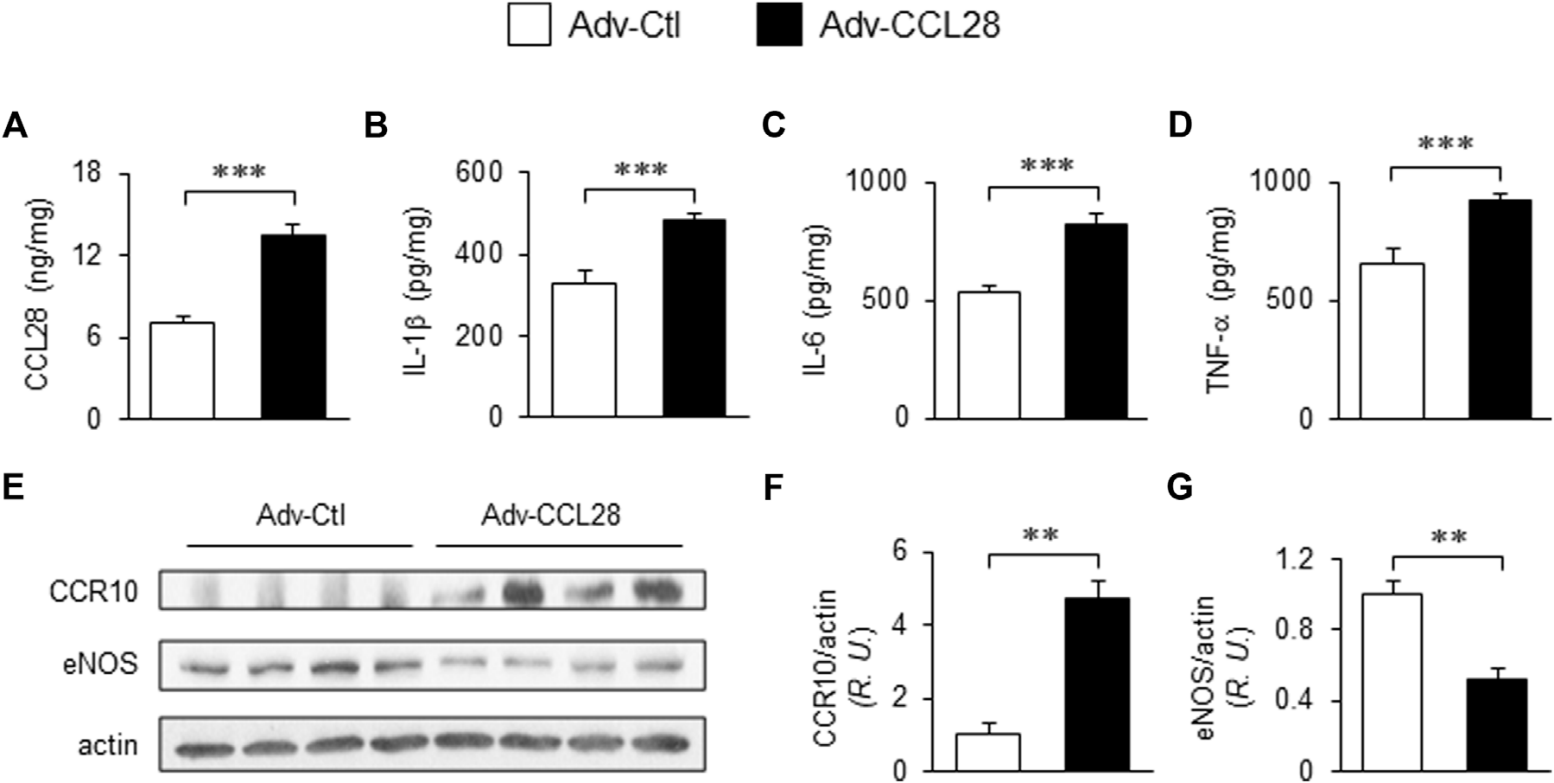
Elevated CCR10 and reduced eNOS expression in dorsal skin of *WT* mice after subcutaneous injection Adv-CCL28. **(A)** Adv-Ctl or Adv-CCL28 (1 × 10^8^/50 µL) were subcutaneously injected into 4 areas of dorsal skin in *WT C57BL/6* mice. Mouse skin wounds were created after 21 days and ELISA measurements from day 7 wound tissues revealed elevated level of CCL28 **(A)**, IL-1β **(B)**, IL-6 **(C)** and TNF-α **(D)**. Values are mean ± SEM, *n* = 6 (***, *p* < .001). **(E)** Western blotting indicated enhanced CCR10 expression **(F)** and reduced eNOS level **(G)** in mice infected with Adv-CCL28. Values are mean ± SEM, *n* = 4–6 (**, *p* < .01). *R.U.*, relative unit.

To investigate whether overexpression of CCL28 *per se*, which we found to be increased in plasma, dorsal skin and adipose tissue of *db/db* mice (Figures 1C–E), results in elevation of CCR10 and reduction of eNOS expression, adenovirus expressing CCL28 (Adv-CCL28) or an empty control vector (Adv-Ctl), was injected subcutaneously in the dorsal skin of *WT* mice. After 3 weeks, the skin was collected for ELISA and Western blot analysis. ELISA measurements indicated enhanced expression of CCL28 (Figure 2A), IL-1β (Figure 2B), IL-6 (Figure 2C), and TNF-α (Figure 2D) following infection with Adv-CCL28, compared with Adv-Ctl. Interestingly, following overexpression of CCL28, Western blotting (Figure 2E) showed significantly increased CCR10 expression (Figures 2E,F) but reduced eNOS level (Figures 2E,G) in the dorsal skin. Taken together, these results suggest that overexpression of CCL28 in dorsal skin results in elevation of CCR10 leading to downregulation of eNOS, along with augmented release of pro-inflammatory cytokines known to contribute to delayed wound healing in diabetic subjects.

### Effect of neutralizing anti-CCL28 Ab on skin wound healing in diabetic *db/db* mice

Next, we investigated whether blockade of excessive CCL28 with a neutralizing antibody affects skin wound healing in diabetic mice. In a pilot dose-finding study, .5 μg/mL anti-CCL28 Ab was topically applied to dorsal skin wounds on both *WT* and *db/db* mice (Supplementay Figure S1A). We observed reduced wound sizes on day 3 (by 36% in *WT* mice; by 12% in *db/db* mice) and day 7 (by 40% in *WT* mice; by 12% in *db/db* mice) following anti-CCL28 Ab treatment, respectively, as compared with IgG control Ab (Supplemental Figure S1B), suggesting that reducing CCL28 level in the dorsal skin may improve wound healing.

**FIGURE 3:**
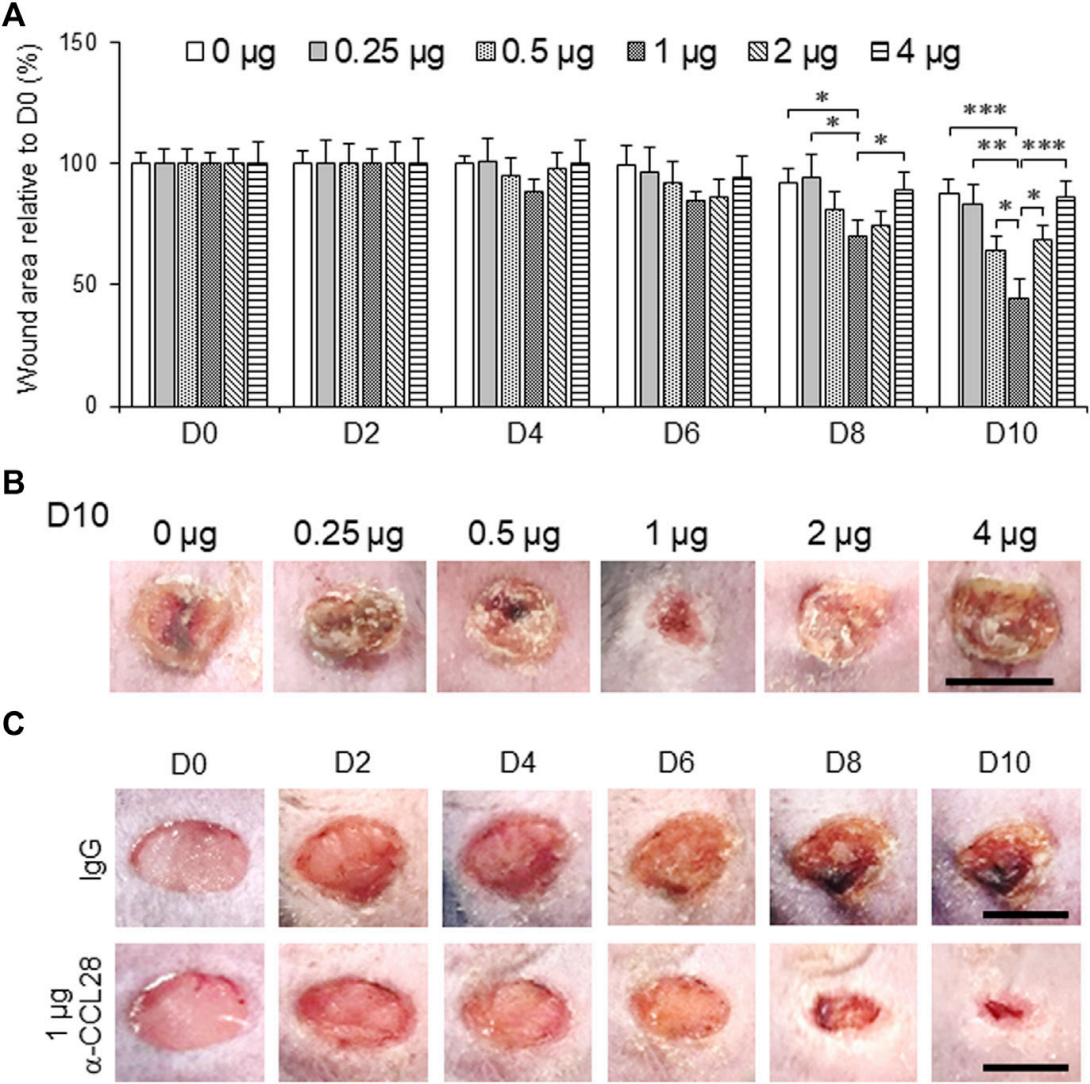
Topical administration of anti-CCL28 antibody (Ab) improves wound healing in diabetic *db/db* mice. **(A)** Effect of different doses of anti-CCL28 Ab on wound healing in *db/db* mice. The wound size on day 0 was normalized to 1. Values are mean ± SEM, *n* = 8 (*, *p* < .05, **, *p* < .01, ***, *p* < .001). **(B)** Photomicrographs of day 10 skin wounds in *db/db* mice after different doses of anti-CCL28 Ab (representative of least 8 independent experiments). Four 5 mm full thickness excisional wounds were made on the mouse dorsal skin, and anti-CCL28 Ab or IgG was applied immediately after punch biopsy. Bar, 5 mm. **(C)** Representative photomicrographs of wounds treated with 1 μg anti-CCL28 Ab or control IgG per wound. Images of the same representative wound from each group were acquired on the days indicated. Bar, 5 mm.

To further investigate the effect of anti-CCL28 Ab on diabetic wound healing, different doses (0–4 μg) of anti-CCL28 Ab were topically administrated to dorsal skin wounds on *db/db* mice (Figure 3). Low doses of .5 and 1 μg anti-CCL28 Ab significantly reduced wound sizes by day 8, whereas higher doses, such as 2 and 4 μg delayed wound healing as shown on day 10 (Figure 3A). Specifically, 1 μg anti-CCL28 Ab was identified as the optimum dose for promoting wound healing (Figures 3A,B), resulting in a nearly completely closed wound by day 10 compared with the same concentration of control IgG (Figure 3C).

### Effect of topical application of anti-CCL28 Ab on eNOS expression and inflammatory environment of the dorsal wounds in *db/db* mice

**FIGURE 4:**
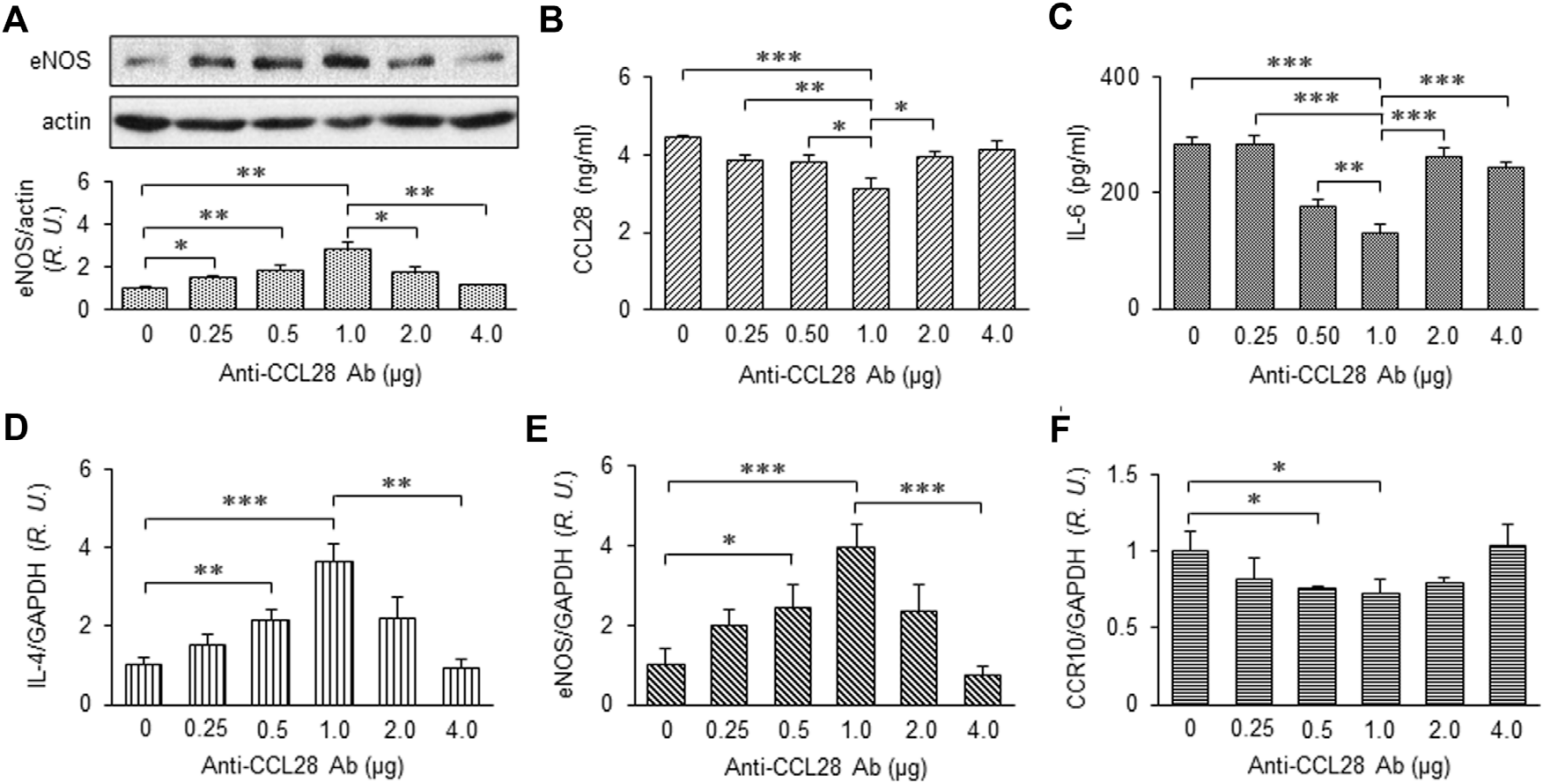
Dose-dependent effect of anti-CCL28 Ab on eNOS expression and anti-inflammatory cytokine production in dorsal wounds of diabetic *db/db* mice. **(A)** eNOS expression level in the mouse wounds 10 days after topical application of .25 to 4.0 mg anti-CCL28 Ab or control IgG. Note eNOS level reached maximum at a dose of 1 μg anti-CCL28 Ab. Values are mean ± SEM, *n* = 4 (*, *p* < .05, **, *p* < .01). ELISA measurement of CCL28 **(B)** and IL-6 **(C)** in the wounds on day 10 at indicated doses of anti-CCL28 Ab. Values are mean ± SEM, *n* = 8 (*, *p* < .05, **, *p* < .01, ***, *p* < .001). mRNA levels of IL-4 **(D)**, eNOS **(E)** and CCR10 **(F)** by real-time RT-PCR in the mouse wounds 10 days after application of different doses of anti-CCL28 Ab. Values are mean ± SEM, *n* = 8 (*, *p* < .05, **, *p* < .01, ***, *p* < .001). *R.U.*, relative unit.

After treatment with different doses of anti-CCL28 Ab, skin wounds from *db/db* mice were collected on day 10 and prepared for further examination. eNOS expression by Western blotting indicated there was a dose-dependent effect of CCL28 Ab (Figure 4A), with maximum eNOS expression observed also at the dose of 1 μg anti-CCL28 Ab per wound. In the wound tissue lysates, levels of CCL28 (Figure 4B) and IL-6 (Figure 4C) determined by ELISA reached a minimum at the dose of 1 μg anti-CCL28 Ab indicating a reduction in inflammation by neutralization of excessive CCL28. In addition, the real-time RT-PCR level of anti-inflammatory cytokine IL-4 (Figure 4D) showed a pattern opposite to that of proinflammatory IL-6 (Figure 4C), but also reached maximum level following 1 μg anti-CCL28 Ab treatment. These data suggest that neutralization of excessive CCL28 reversed the inflammatory environment and facilitated wound healing in diabetic mice. In addition, mRNA level of eNOS (Figure 4E) paralleled that of eNOS protein (Figure 4A), with maximum levels achieved with 1 μg anti-CCL28 Ab. Interestingly, CCR10 level was also maximally reduced at the same 1 μg dose of anti-CCL28 Ab. Taken together, these data suggest that depletion of excessive CCL28 by Ab neutralization led to enhanced eNOS expression and reduced CCR10 level, along with a switch from a proinflammatory to anti-inflammatory environment that collectively are thought to have contributed to improved wound healing in diabetic animals.

### Topical application of optimum dose of anti-CCL28 Ab enhanced eNOS/NO levels and microvessel density in *db/db* mouse dorsal skin wounds

**FIGURE 5:**
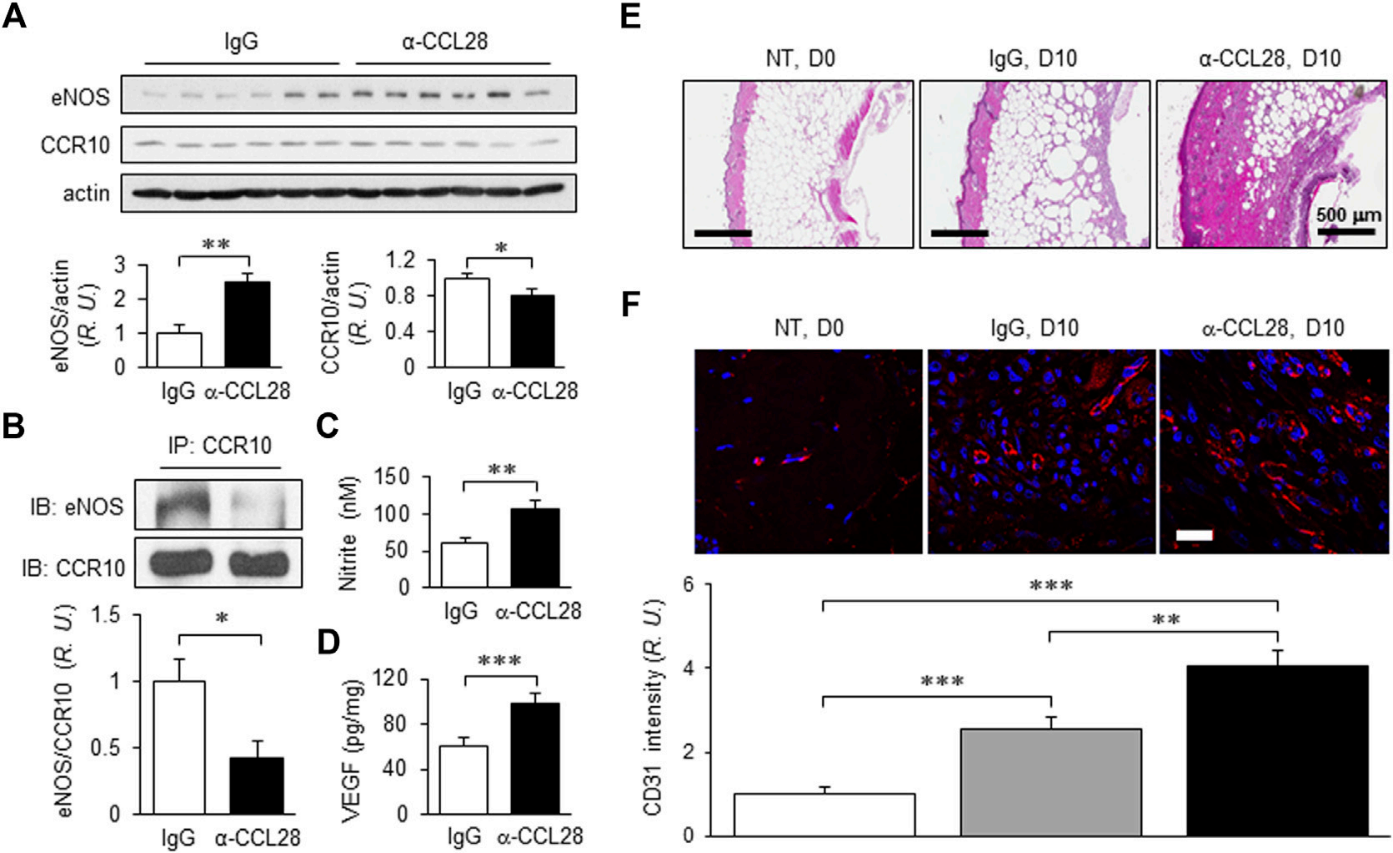
NO and VEGF levels, regrowth of the dermal layers, and microvessel density of day 10 *db/db* mouse dorsal skin wounds following treatment with 1 μg anti-CCL28 Ab. **(A)** Elevated eNOS expression and reduced CCR10 level in day 10 mouse wounds after application of 1 μg anti-CCL28 Ab. eNOS and CCR10 levels were normalized to actin; the IgG control group was taken as 1. Values are mean ± SEM, *n* = 6 (*, *p* < .05, **, *p* < .01). **(B)** Reduction of eNOS-CCR10 interaction in dorsal wounds of *db/db* mice 10 days after treatment with 1 μg anti-CCL28 Ab. The wounds were collected and anti-CCR10 Ab was added to the tissue lysates. The pull-down samples were assessed by Western blotting with anti-eNOS Ab (top panel) and anti-CCR10 Ab (bottom panel). The ratio between eNOS and CCR10 in the IgG treated wounds were normalized to 1. Values are mean ± SEM, *n* = 6 (*, *p* < .05). *R.U.*, relative units. Elevation of nitrite **(C)** detected by Griess Reagent and VEGF level **(D)** by ELISA measurement from day 10 mouse wounds following treatment with 1 μg anti-CCL28 Ab or control IgG. Values are mean ± SEM, *n* = 4–6 (**, *p* < .01. ***, *p* < .001). **(E)** Representative H&E staining showing extent of regrowth of *epidermis*, dermis, and hypodermis in skin wounds in *db/db* mice treated with 1 μg anti-CCL28 Ab, as compared to control IgG. Bar, 500 μm. **(F)** Microvessel density as indicated by immunostaining of CD31 (red) in *db/db* mouse wounds 10 days after anti-CCL28 Ab treatment, compared to control IgG. Representative of 6 independent experiments. Bar, 20 μm. The density of CD31 immunostaining was qualified with Zeiss Zen and ImageJ software, and day 0 with no treatment (NT) was set as 1. Values are mean ± SEM, *n* = 6 (**, *p* < .01, ***, *p* < .001).

With the optimum dose of 1 μg anti-CCL28, day 10 eNOS expression level determined by Western blot analysis was about 2.5-fold higher than that observed in control IgG treated *db/db* mouse wounds, while CCR10 level decreased by about 20% (Figure 5A). Previously, we reported that CCR10 binding to eNOS increases following addition of its ligand CCL28 (Chen et al., 2020; Chen et al., 2022) to human ECs. Consistent with this observation, inhibition of CCL28 signaling in diabetic skin wounds with a neutralizing anti-CCL28 Ab reduced the interaction between CCR10 and eNOS in mouse wounds, as assessed by Co-IP, as compared to IgG control treated wounds (Figure 5B). Interestingly, in the anti-CCL28 Ab treated wounds, we observed elevated levels of both NO (nitrite; Figure 5C) and VEGF (Figure 5D). Furthermore, anti-CCL28 treatment resulted in a thicker *epidermis*, as well as dermis and hypodermis, in day 10 skin wounds as observed in H&E stained wound sections (Figure 5E). Microvessel density, indicated by CD31 immunohistochemistry, was also significantly enhanced in anti-CCL28 Ab treated wounds as compared with IgG control (Figure 5F). These results suggest neutralization of CCL28 reduces CCR10-eNOS interaction, thereby increasing eNOS/NO level, which may have further lead to an increase in VEGF production as well as angiogenesis that collectively facilitated diabetic wound healing.

eNOS-deficient mice exhibit impaired wound healing and angiogenesis defects (Lee et al., 1999) and thus we assessed levels of CCL28 and proinflammatory cytokines IL-1β, IL-6 and TNF-α by ELISA of wound lysates. As shown in Supplementary Figure S2A-D, CCL28 and cytokines were significantly elevated in the dorsal skin of eNOS^*−/−*^ mice. In addition, CCR10 mRNA level was significantly increased (Supplementary Figures S2E). Following treatment with 1 μg/mL anti-CCL28 Ab, skin wound size on eNOS^*−/−*^ mice was significantly reduced starting on day 3, and by day 10 was 46% smaller than those in the IgG control group (Supplementary Figures S2F-S2G). These results indicate anti-CCL28 has both eNOS dependent and independent effects on wound healing. Based on these findings, neutralization of excessive CCL28 appears to have at least two effects: 1) reduced expression of pro-inflammatory cytokines and CCR10, and 2) blockade of CCR10 activation and its interaction with eNOS in endothelial cells, resulting in upregulation of eNOS/NO level and VEGF production, both of which are thought to facilitate wound healing in diabetic mice.

**FIGURE 6:**
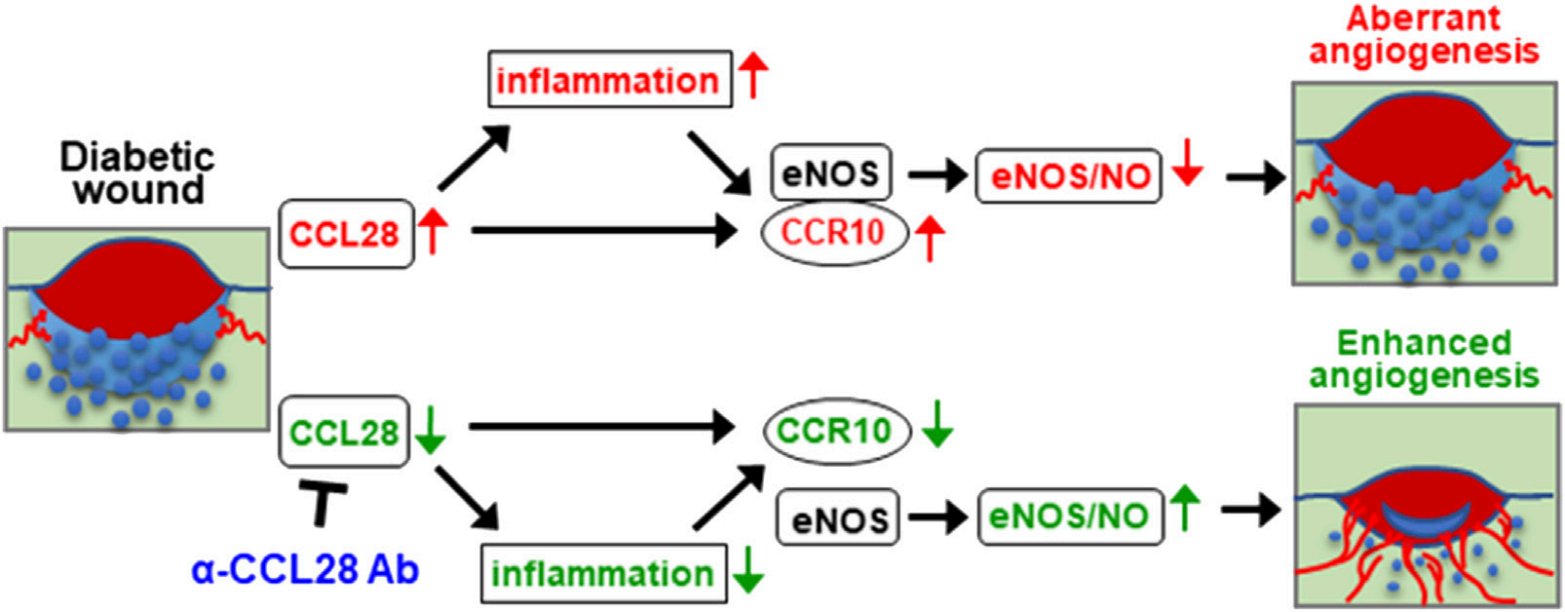
Schematic hypothesis: Excessive CCL28 through its interaction with CCR10 in subjects with diabetes results in aberrant angiogenesis and delayed wound healing. In diabetic skin wounds, an excessive level of chemokine CCL28 induces inflammation and sustained activation of CCR10 receptor, increasing is binding to eNOS which inhibits eNOS activity and downregulates expression leading to reduce NO production, aberrant or unproductive angiogenesis, and delayed wound healing. Neutralization of excessive CCL28 with anti-CCL28 Ab reduces inflammation and blocks intracellular CCR10-eNOS interaction in ECs, thereby restoring eNOS expression and NO production, enhancing wound angiogenesis and regrowth of the *epidermis*, dermis, and hypoderms, thereby improving diabetic skin wound healing.

Based on the above findings, upregulation of chemokine CCL28 in the *db/db* diabetic mouse promotes inflammation and sustained CCR10 receptor activation and binding to eNOS in endothelial cells. Excessive activation of CCR10 on endothelial cells inhibits eNOS activity and downregulates eNOS expression and NO production leading to a further increase in inflammation, decrease in angiogenesis, and delay in wound healing. We propose that neutralization of excessive CCL28 with 1 μg anti-CCL28 Ab reduces and perhaps normalizes CCL28 signaling in endothelial and non-endothelial CCR10 expressing cells resulting in reduced inflammation and eNOS downregulation, thereby improving wound healing in diabetic mice by NO dependent and NO independent anti-inflammatory and proangiogenic mechanisms (Figure 6).

## Discussion

In this paper, a novel strategy to improve chronic dorsal skin wound healing was evaluated in obesity-induced diabetic *db/db* (*Lepr* ^*db+/db+*^) mice. Topical administration of anti-CCL28 Ab to diabetic mouse skin wounds resulted in reduced inflammation, elevated angiogenesis, regrowth of the *epidermis*, dermis and hypodermis, and improved wound healing. We observed excessive expression of CCL28 in plasma and skin samples from biopsies of both human patients with T2DM and diabetic animals. In addition, elevated CCL28 production was also observed in conditioned medium of white adipose from diabetic animals. Furthermore, overexpression of CCL28 *via* Adv-CCL28 in dorsal skin of *WT* mice escalated inflammation as well as reduced eNOS level leading to delayed wound healing. Thus, we conducted neutralization studies using anti-CCL28 Ab which showed dose-dependent effects on the levels of pro-inflammatory cytokines, CCR10, and eNOS in dorsal skin wounds of *db/db* mice. At the optimum dose of 1 μg anti-CCL28 Ab per wound, improved wound healing was observed along with a reduction in inflammation and CCR10-eNOS interaction, and upregulation of eNOS/NO and VEGF levels leading to enhanced microvessel density as well as regrowth of the dermal layers. These findings generate novel insights into the role of CCL28/CCR10 signaling in pathological angiogenesis, inflammation, and delayed skin wound healing associated with T2DM and potentially may lead to novel therapeutic strategies for DFUs.

Wound healing is a complex and dynamic process consisting of four overlapping phases that happen in a precise and regulated manner: hemostasis, inflammation, proliferation, and maturation (Gosain and DiPietro, 2004; Mathieu, 2006). DFUs take longer to heal because of improper integration of angiogenesis and inflammation as well as disordered immune responses (Xu et al., 2013; Singh et al., 2015). The uncontrolled or prolonged inflammatory responses frequently observed in diabetic wounds leads to the impairment of subsequent phases of the wound healing process, and thus implicated as a contributor to ulceration (Wong et al., 2015; Eming et al., 2017). Diabetic ulcers are characterized by a chronic inflammatory state primarily manifested by imbalances in pro- and anti-inflammatory cytokines (Pierce, 2001). Dysfunctional macrophage efferocytosis has also been reported to impair resolution of inflammation in the wounds of diabetic *db/db* mice (Khanna et al., 2010).

CCL28 is upregulated by the proinflammatory cytokine IL-1, bacterial flagellin, and *n*-butyrate in human colon epithelium (Ogawa et al., 2004), and by IL-1β, TNF-α and IL-17A in decidual stromal cells (DSCs) (Sun et al., 2013). Human recombinant CCL28 promoted DSC apoptosis, which was eliminated by pretreatment with neutralizing anti-CCL28 Ab (Sun et al., 2013). We previously observed protein expression of CCL28 and CCR10 to be upregulated by LPS and pro-inflammatory cytokines such as TNF-α and IL-6 in monocytes and macrophages isolated from patients with rheumatoid arthritis (RA). Neutralization of CCL28 in RA synovial fluid (SF) or blockade of CCR10 on human endothelial progenitor cells (EPCs) significantly reduced SF-induced endothelial migration and capillary formation indicating that CCL28/CCR10 is involved in pathological RA angiogenesis (Chen et al., 2015).

On the one had, in different tissues and plasma samples from patients with T2DM and diabetic mice, we showed there was excessive levels of CCL28 (Figure 1A; Chen et al., 2022). We demonstrate that overexpression of CCL28 *via* injection of Adv-CCL28 in *WT* mouse skin caused a burst in expression of pro-inflammatory cytokines and CCR10, reduced eNOS expression, and delayed wound healing. Similar results were obtained when *WT* mice were injected with Adv-CCR10 (Chen et al., 2022) suggesting excessive CCL28/CCR10 signaling plays an important role in induction of inflammation and aberrant angiogenesis associated with chronic or non-healing skin wounds. G-protein coupled receptors (GPCRs) are known to be involved in the regulation of NF-κB and inflammation (see reviews: Ye, 2001; Fraser, 2008; Yu and Brown, 2015). However, the mechanisms by which CCL28/CCR10 promotes activation of NF-κB and inflammation in diabetic wounds is unclear and requires further investigation. On the other hand, reduced migration of IgA ASC cells and dysfunctional antimicrobial activity were observed in the colon of CCL28-deficient mice (Matsuo et al., 2018). In addition, abnormal neutrophil recruitment and activation resulted in increased susceptibility to infection in the gut by *Salmonella* in CCL28 knockout mice (Burkhardt et al., 2019). Here, we show that application of 1 μg or less anti-CCL28 Ab per wound, there is a reduction in inflammation and increased levels of anti-inflammatory cytokines and eNOS thought to improve diabetic wound healing. However, higher levels (>1 μg per wound) resulted in detrimental effects on the healing process. When higher doses of anti-CCL28 Ab (2 and 4 ug/wound) were applied topically to fresh wounds, healing time was delayed, there was an increase in the level of pro-inflammatory factor IL-6, a decrease in the level of eNOS, and reduced microvessel density. Similar observations of delayed skin wound healing were made in the *CCR10* ^*−/−*^ mouse model (Chen et al., 2022). These results suggest that high or low levels of CCL28 can lead to pathological consequences, and that tight regulation of CCL28 and CCR10 expression are important for maintaining homeostasis in the skin microenvironment.

It was previously reported that deficits in the mouse skin wound healing response due to diet-induced obesity could be overcome by increasing NO production *via* overexpression of eNOS (Sansbury et al., 2012). MMP-2 activity, hydroxyproline (OHP) content, and wound breaking strength were significantly increased by the NO donor molsidomine, which partially reversed impaired healing in diabetic rats (Witte and Barbul, 2002; Witte et al., 2002). A statin-loaded tissue engineered scaffold (TES) that promotes eNOS expression and NO synthesis in and around the regenerating tissues in a rat model of diabetes was also shown to accelerate vascularization and elevate blood supply, thereby decreasing wound healing time (Yang et al., 2016). Further, recombinant adeno-associated virus for expressing angiopoietin-1 (rAAV-Ang-1) increased eNOS expression and augmented nitrate wound content, VEGFR-2 immunostaining and protein expression, and improved wound repair in *db/db* mice by inducing angiogenesis in a VEGF-independent manner (Bitto et al., 2008). Acarbose (α-glucosidase inhibitor), a widely used oral glucose-lowering drug exclusively for T2DM, increased circulating EPC number and NO production, thus also exhibiting potential as a therapeutic for promoting wound healing and improving angiogenesis in *db/db* mice, which may be related to activation of Akt/eNOS signaling (Han et al., 2017).

We recently reported that blockade of CCR10-eNOS interaction in ECs *via* a 7 amino acid eNOS-derived cell permeable CCR10 binding domain peptide (Myr-CBD7) resulted in upregulation of eNOS/NO level, promoted a more favorable anti-inflammatory environment, stimulated angiogenesis, and accelerated wound closure in diabetic mice (Chen et al., 2022). Here we demonstrate that neutralization of excessive CCL28 not only reduced inflammation but also CCR10-eNOS interaction in diabetic wounds, thereby enhancing eNOS/NO level, VEGF production, microvessel density, and wound healing in the obesity-induced mouse model of type 2 diabetes. Whereas Myr-CBD7 targets and disrupts eNOS-CCR10 interactions in endothelial cells, topically applied anti-CCL28 mAb neutralizes CCL28 in the wound microenvironment and thereby would reduce CCL28-activated CCR10 as well as CCR3 signaling in multiple cell types. It will be important to assess the relative efficacy of Myr-CBD7 and anti-CCL28 Ab treatments, alone and in combination, in subsequent investigations of diabetic skin wound healing in *db/db* mice, in larger animal models such as the high fat-fed mini-pig, as well as patients with T2DM to determine if Myr-CBD7 or anti-CCL28 Ab are effective pharmacological approaches for improving non-healing diabetic skin wounds such as DFUs.

In conclusion, we have demonstrated that an excessive level CCL28, *via* activation of its receptor CCR10, leads to a reduction in eNOS/NO and elevation in proinflammatory cytokines resulting in less angiogenesis and delayed skin wound healing time in diabetic animals. Neutralization of excessive CCL28 with anti-CCL28 Ab provides a potential therapeutic option for treatment of diabetic skin wounds such as DFUs by increasing NO production and reducing inflammation, most likely by normalizing CCL28 signaling in multiple cell types.

## Data Availability

The original contributions presented in the study are included in the article/Supplementary Material, further inquiries can be directed to the corresponding authors.
