# Supplementary material for: Neutralization of excessive CCL28 improves wound healing in diabetic mice
Source: Front Pharmacol. 2023 Jan 13;14:1087924. doi: 10.3389/fphar.2023.1087924 (PMC9880283; doi:10.3389/fphar.2023.1087924)
Supplement: Supplementary file 1 [file DataSheet1.docx]

**Supplemental Materials**

**Supplemental Figure S1**


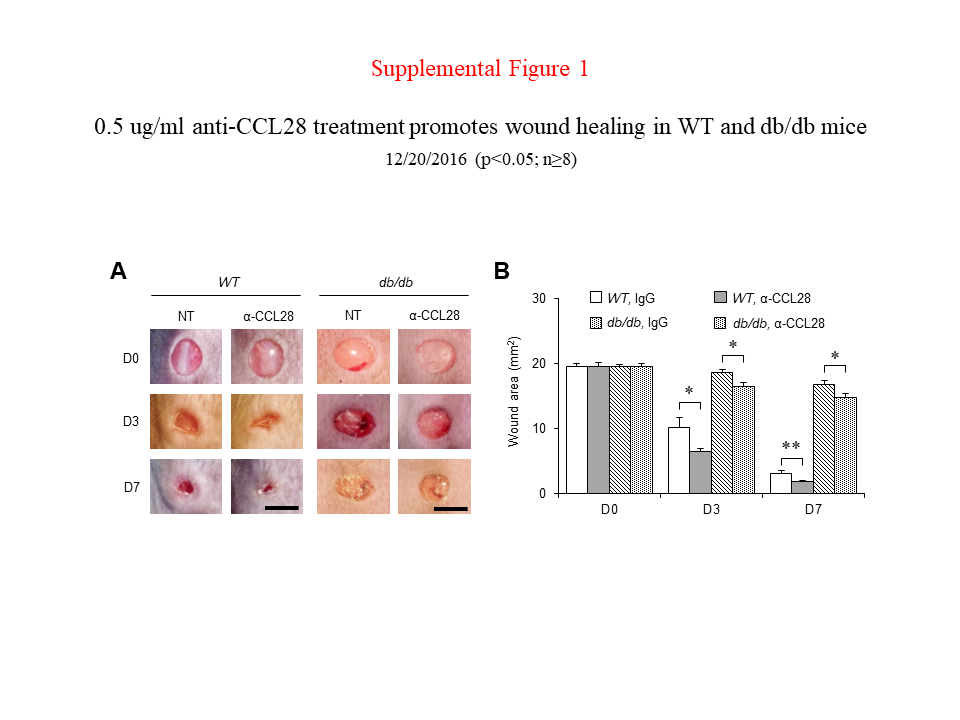


**Supplemental Figure S1. Effect of topical administration of anti-CCL28 Ab on dorsal skin wound healing time in *WT* and diabetic *db/db* mice.** (**A**) Representative photomicrographs of day 0, 3 and 7 wounds following treatment with 0.5 μg anti-CCL28 Ab or control IgG per wound. Four 5 mm full thickness excisional wounds were made on the mouse dorsal skin and anti-CCL28 Ab or IgG was applied immediately after wounding. Images are of the same wound taken before (day 0) and after treatment; Bar, 5 mm. (**B**) Wound sizes in *WT* and *db/db* mice after application of 0.5 μg anti-CCL28 Ab or IgG per wound. Values are mean ± SEM, n=8-12 (*, *p*<0.05, **, *p*<0.01 by one-way ANOVA).

**Supplemental Figure S2**


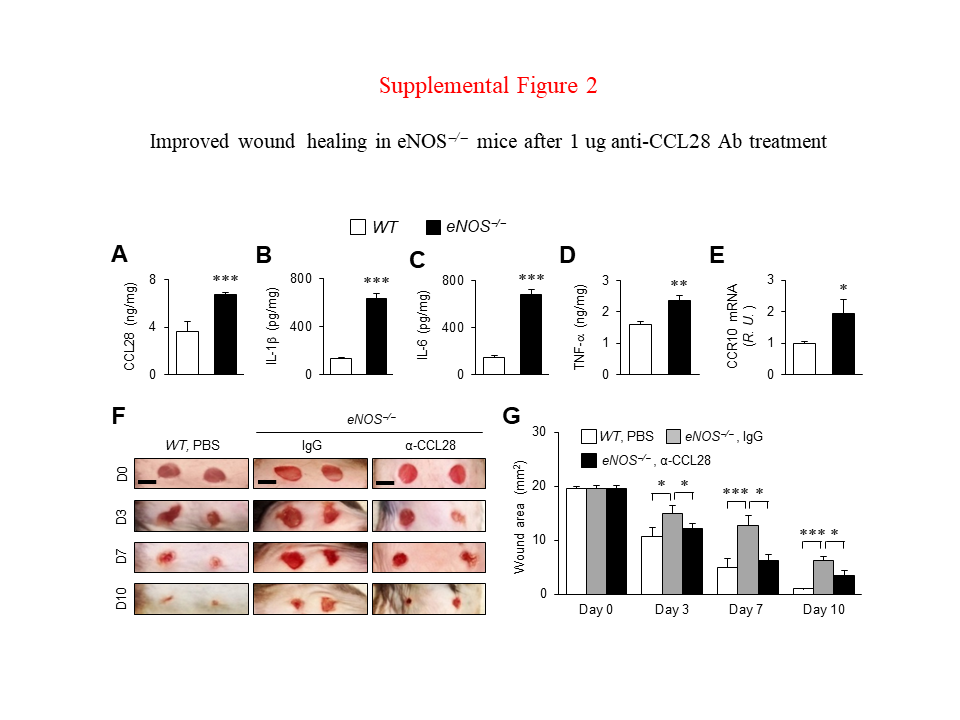


**Supplemental Figure S2. Topical administration of anti-CCL28 Ab reduced skin wound sizes in *eNOS^−/−^* mice.** ELISA measurement indicated elevation of CCL28 (**A**), pro-inflammatory cytokines IL-1β (**B**), IL-6 (**C**) and TNF-α (**D**), as well as increased mRNA level of CCR10 (**E**) by real-time RT-PCR in dorsal skin of *eNOS^−/−^* mice. Values are mean ± SEM, n=8-12 (*, *p*<0.05, **, *p*<0.01, ***, *p*<0.001 by Student’s t-test). (**F**) Representative photomicrographs of wounds treated with 1 μg anti-CCL28 Ab or control IgG per wound. Four 5 mm full thickness excisional wounds were made on the mouse dorsal skin, and anti-CCL28 Ab or IgG was applied immediately after wound punching. For *WT* mice, PBS was topically applied on the wounds. Images are of the same wounds taken at indicated days. Bar, 5 mm. (**G**) Wound area on *eNOS^−/−^* mice after application of 0.5 μg anti-CCL28 Ab or control IgG per wound as compared to PBS-treated *WT* mice. Values are mean ± SEM, n=4-10 (*, *p*<0.05, ***, *p*<0.001 by ANOVA).
